# Sleep and recovery in physicians on night call: a longitudinal field study

**DOI:** 10.1186/1472-6963-10-239

**Published:** 2010-08-15

**Authors:** Birgitta Malmberg, Göran Kecklund, Björn Karlson, Roger Persson, Per Flisberg, Palle Ørbaek

**Affiliations:** 1 Occupational and Environmental Medicine, Dept of Laboratory Medicine, Lund University, Lund, Sweden; 2 Stress Research Institute, University of Stockholm, Stockholm, Sweden; 3 National Research Centre for the Working Environment, Copenhagen, Denmark; 4 Department of Anaesthesiology and Intensive care, Skåne University Hospital, Lund, Sweden

## Abstract

**Background:**

It is well known that physicians' night-call duty may cause impaired performance and adverse effects on subjective health, but there is limited knowledge about effects on sleep duration and recovery time. In recent years occupational stress and impaired well-being among anaesthesiologists have been frequently reported for in the scientific literature. Given their main focus on handling patients with life-threatening conditions, when on call, one might expect sleep and recovery to be negatively affected by work, especially in this specialist group. The aim of the present study was to examine whether a 16-hour night-call schedule allowed for sufficient recovery in anaesthesiologists compared with other physician specialists handling less life-threatening conditions, when on call.

**Methods:**

Sleep, monitored by actigraphy and Karolinska Sleep Diary/Sleepiness Scale on one night after daytime work, one night call, the following first and second nights post-call, and a Saturday night, was compared between 15 anaesthesiologists and 17 paediatricians and ear, nose, and throat surgeons.

**Results:**

Recovery patterns over the days after night call did not differ between groups, but between days. Mean night sleep for all physicians was 3 hours when on call, 7 h both nights post-call and Saturday, and 6 h after daytime work (p < 0.001). Scores for mental fatigue and feeling well rested were poorer post-call, but returned to Sunday morning levels after two nights' sleep.

**Conclusions:**

Despite considerable sleep loss during work on night call, and unexpectedly short sleep after ordinary day work, the physicians' self-reports indicate full recovery after two nights' sleep. We conclude that these 16-hour night duties were compatible with a short-term recovery in both physician groups, but the limited sleep duration in general still implies a long-term health concern. These results may contribute to the establishment of safe working hours for night-call duty in physicians and other health-care workers.

## Background

Studies of physicians have shown that night-call duty with long work hours, restricted sleep, stress, time pressure, and high demands may cause impaired performance and adverse effects on subjective health [1-4]. Working schedules involving long working hours also seem to have a negative influence on caregivers' decision-making capabilities, which may have a profound impact on patients' safety [5,6]. Although sleep is crucial to recovery and survival, there is limited knowledge about the effects of night-call duty on sleep duration, sleep quality, and recovery time. One might assume effects similar to those from classic rotating shift work, but the irregular character of night-call schedules and the longer shifts make comparison difficult. In a study from 1990, Åkerstedt and co-workers monitored six physicians on night call using ambulatory EEG, which revealed a considerable sleep deficit with only 3 hours of night sleep [7]. However, the requirements for on-call work have changed over the past few decades, with a tendency for more intense night-call work, often resembling a full night shift, but with a successive shortening of shift lengths in return. These changes have made the effects on sleep difficult to anticipate. It has been well documented that night shifts of 24 hours or longer are, in most cases, detrimental to both subjective health and performance [1,3,5,8]. However, studies focusing on shorter night schedules are scarce and there is considerable controversy regarding the optimal length of a night-call shift. Therefore, it is important to study these aspects of sleep and performance in current night-call schedules to obtain empirical evidence on which to base sound shift scheduling [9,10]. Not only the length of the night-call shift, but also its intensity and mentally demanding character, may be assumed to affect sleep and recovery. Given the characteristics of their work, anaesthesiologists are reported to have a higher mental workload during night duty compared with many other physician groups working on call [11-14]. Indeed, during recent years, occupational stress and impaired well-being in anaesthesiologists have been frequently addressed in the scientific literature [13,15,16]. For the anaesthesiologist on call, the cognitive and emotional load of constantly handling patients with life-threatening conditions, which requires fast and accurate action, may have a greater impact on their sleep and recovery compared with other specialists.

A controlled longitudinal field study was performed at a university hospital. The present study was part of a large field study of physiological restitution after night-call duty, involving several sub-studies, each focusing on different aspects and potential negative health effects of the anaesthesiologists' work schedules: the impact on metabolic factors has been published earlier [17]. The participating clinics agreed that if the results indicated insufficient recovery, the work schedules would be adjusted. Hence, it was expected a priori that sleep duration and quality of sleep would be negatively affected in physicians on night call, especially for anaesthesiologists. The primary aim of this study was therefore to evaluate whether a 16-hour night-call schedule allowed for sufficient recovery in anaesthesiologists compared with other physician specialists handling less life-threatening conditions when on call.

## Methods

### Participants

We enrolled two groups of physicians. The first group consisted of anaesthesiologists (ANEST; n = 19). The second group consisted of paediatricians and ear, nose, and throat (ENT) surgeons (PENT; n = 17). The two groups had different work characteristics when on call (see below). Originally, only paediatricians were planned to constitute the control group; during recruiting, however, fewer physicians were available in clinical work than expected, thus we chose to augment the control group by including ENT surgeons. The physicians in both groups worked at the same university hospital and had on-call duties that involved working recurrent night shifts. All physicians holding the above-mentioned positions were asked to participate. The participation rate was 19/24 (79%) in the ANEST group and 17/25 (68%) in the PENT group. Self-report data missing for one anaesthesiologist and technical errors in three of the sleep registrations in this group forced us to discard data for four participants, hence the final ANEST sample consisted of 15 anaesthesiologists. Demographic data collected in the baseline questionnaire are presented in Table 1. Data from a baseline inventory showed that the family situations were almost identical in the two groups of physicians and that there were no cases of cardiovascular disease, diabetes, insomnia, or sleep apnoea syndrome. From data obtained in a previously published sub-study we also knew that they shared a low to moderate alcohol consumption, normal liver function, serum insulin, plasma glucose, and blood lipids [17]. Baseline data revealed no between-group differences concerning subjective reports on sleep sufficiency and recovery from work. In the pooled group of physicians, 17% reported the time generally needed for sufficient recovery from night call to be one night's sleep, 73% reported a need for two nights' sleep, and 10%, reported needing three nights' sleep or more. Morning diurnal type was reported by 37% and evening type by 63% [18]. The reported mean need of sleep for feeling well rested in the morning was 7:28 hours (SD = 44 min).

**Table 1:** Demographic data for participating anaesthesiologists (ANEST) and paediatricians and ENT surgeons (PENT).

	ANEST (n = 15)	PENT (n = 17)^a^
Median age (range)	43 (37-55)	37 (26-45)
Mean BMI in kg/m^2 ^(SD)	24 (2.9)	22 (1.9)
Median years' experience of night call (range)	10 (4-27)	11 (0-19)
Women (%)	6 (40)	8 (47)
Smokers (%)	0	0
*Position:*		
Consultants (%)	6 (40)	2 (13)
Registrars (%)	6 (40)	4 (27)
Residents (%)	3 (20)	9 (60)
*Social status *		
Single (%)	0	1(7)
Living with other adult, but no children (%)	3 (20)	3 (20)
Living with other adult and children (%)	12 (80)	11 (73)
Living with children, but without other adult (%)	0	0
*Work/home interface*		
Worries about family matters influence focus on work (%)	5 (33)	5 (33)
Regular overtime (≥ once a week) (%)	7 (47)	6 (40)

^a ^For two participants only data on age, gender, and BMI were available.

### Ethics

All participants gave their written informed consent and the study was approved by the Ethics Committee at Lund University (LU 732-01).

### Working conditions

The reason for choosing the two groups ANEST and PENT as participants was motivated by that the characteristics of their working conditions, at the time of data sampling, implied a clear between-group exposure contrast in activity levels and mental demands. All participants had strictly hospital-bound work on call, but with less focus on life-threatening conditions for the PENT group. During night-call duty, the ANEST group took care of patients' vital functions in major traumas, cardiac arrest, and acute operations. They were also in charge of post-operative ward and the intensive care unit, handling patients with vital organ failure. All participating physicians could expect to have a high workload on call, but the ANEST group also had to focus generally on life-threatening conditions. Furthermore, they differed from the PENT group in that they provided service to the whole hospital and facilitated the work of several other physicians caring for severely ill patients [11]. In contrast, the PENT group worked at the emergency ward taking care of acute cases in their speciality, but they did not have the kind of service and facilitation duties required of the ANEST group. The specific information on working conditions was collected through interviews with the specialists and by scrutinizing all working schedules. For all physicians, ordinary daytime work was performed from 08:00 to 16:30. Night-call duty started at approximately 16:00 and lasted for about 16 hours (until around 08:00 the next day). All participants had on average three night-call duties per month and at least one day off after each night call, but work schedules differed somewhat between specialities. The ANEST group and paediatricians in the PENT group had separate night-call weeks every fourth to sixth week, during which they worked two to three nights. In the PENT group the ENT surgeons had single nights on call every second or third week, and in contrast to the two other specialities, they generally had a normal working day directly before night call. There were no scheduled breaks during on-call duty, but breaks were allowed for meals, rest, or even sleep if there were no patients in need of attention; on-duty rooms were always available.

### Rating scales and activity measures

*Karolinska Sleep Questionnaire (KSQ) *was used to assess participants' habitual quality of sleep [19]. This questionnaire comprises 15 items measured on a 5-point scale, including difficulties falling asleep, disturbed sleep, too little sleep (< 6 h), repeated awakenings, premature awakening, exhaustion at awakening, not well rested on awakening, difficulties awakening, sleepiness during the day, nodding off at work, nodding off during leisure time, sleep quality, sufficient sleep, nightmares, and heavy snoring. The scores/response alternatives are: 1 = always/every day, 2 = mostly/several days a week, 3 = sometimes/several times a month, 4 = seldom/a few times a year, 5 = never. Three indices were calculated from 12 of the 15 items: "disturbed sleep", "sleepiness index," and "awakening index," with 5 as the most positive score. Three participants had missing data for KSQ.

*Karolinska Sleepiness Scale (KSS) *was used to assess current sleepiness and was completed at bedtime. This scale has been used in many studies and has been validated against EEG parameters [20]. This is a 9-point scale with the following verbal anchors: 1 = very alert, 3 = alert, 5 = neither alert nor sleepy, 7 = sleepy, but with no difficulty staying awake and 9 = very sleepy, fighting against sleep, requiring great effort to stay awake. The intermediate values are also used, but they have no labels.

*Mental fatigue *was assessed on a scale similar to the KSS with the following verbal anchors: 1 = very alert, 3 = alert or energetic, 5 = neither fatigued nor alert, 7 = fatigued but not strained, and 9 = very fatigued, exhausted, and incapable of any mental strain. The intermediate values were also used, but they had no labels. In a previous study, individuals with a high "burnout" score also scored high on the mental fatigue scale [21]. In addition, the mental fatigue scale has demonstrated higher levels of fatigue during workdays compared to weekends, a time-of-day profile (peaking in the evening), and elevated levels of fatigue in association with early morning shifts [21,22].

*Karolinska Sleep Diary (KSD) *was used to assess daily variations in the subjective aspects of sleep and recovery as experienced in the morning [23]. This questionnaire has been validated against polysomnography and shows good correlation with objective EEG sleep measures (e.g. amount of slow-wave sleep and sleep efficiency) [23-25]. The questionnaire consists of several items concerning the previous night's sleep and aspects of sleep recovery. All variables are scored from 1 to 5, where 5 is the most positive. A Sleep Quality Index (SQI) was constructed from the mean scores on the questions regarding "restless sleep", "ease of falling asleep," "sleep quality," and "sleep through the night." The SQI ranged from 1 (low quality) to 5 (high quality). The questions on "sufficient sleep," "ease of awakening," and "feeling well rested," reflecting aspects of sleep and recovery, were studied as single items. Sufficient recovery, in subjective terms, was defined as the score on KSD for feeling well rested after a Saturday night's sleep.

*Actigraphy *was used to record sleeping and waking activity (Actiwatch, Cambridge Neurotechnology Ltd, UK) [26]. The Actiwatch device is a wrist-worn accelerometer that measures wrist activity, which has proved to be a good proxy for EEG-registered sleep. The output sleep scores have a high correspondence with polysomnographically recorded sleep and are also validated for documenting longitudinal changes in sleep patterns [27,28]. The watch was calibrated and set to personal computer (PC) time and an epoch length of 0.5 minutes was chosen. A medium sensitivity was applied. The participants wore the Actiwatch (AW) on the non-dominant wrist during the whole study period, except when taking a shower, during strenuous physical training, and when they had to work in aseptic conditions. In addition, the participants kept a separate *log-book *for daily notes on bedtime, rising time, and special circumstances or events of significance for AW monitoring. The latter could be an emergency alarm, physical training, or removal of the AW. Participants were also instructed to press the event-button at bedtime and upon rising. Start and end times for night sleep were determined manually, with sleep onset defined as the first period of 5 minutes or more of immobility according to actigraphic recording in the evening and sleep end as the stable return of measurable actigraphic activity for at least 10 minutes in the morning. Although the AW sleep-wake registrations were used as primary information, the notes in the log books concerning special circumstances at bedtime upon rising and at AW removal were used as complementary information to define the most accurate start and end points for sleep analysis. All periods accepted as sleep were also checked against the sleep logs. In the majority of participants the correspondence between sleep log data and AW data was excellent. The Actiwatch Sleep Analysis program (version 5.48) was used for data scoring. Periods of waking and sleeping between sleep start and sleep end were scored automatically by the Actiwatch algorithm, which was also used to calculate total sleep time and sleep efficiency (% of time during the period from sleep start to sleep end spent actually sleeping). Bedtime and rising time were missing from some participants' log books, which is why the customary way of calculating sleep efficiency (which includes sleep latency) could not be used. Instead it was measured from sleep start to sleep end as recorded by the AW. A separate analysis of day-time naps was performed on the first post-call day using the same methodology as for the night sleep analyses.

### Study design and procedures

The study was designed to answer questions about sleep and recovery in relation to night-call duty. At the beginning of the study, participants completed a baseline inventory containing the KSQ and other health- and background data in order to characterize the participants. Next, the monitoring period for studying the effects of daytime work, night call, and post night-call on the various measures was chosen to fit each individual's work schedule. Participants were then continuously monitored for 10 to 22 days, during both work and leisure time, depending on their individual schedule. Requirements for several substudies and the specific personal night-call schedules directed the total length of monitoring period for each participant.

The KSS and mental fatigue-scores were completed at bedtime in the evening and the KSD was completed in the morning soon after awakening. Because bedtimes and rising times were quite different when participants were on night call, and this could lead to both compliance problems and difficulties in interpretation, participants were instructed to refrain from completing the KSS and KSD when they were on night call.

**Figure 1:**
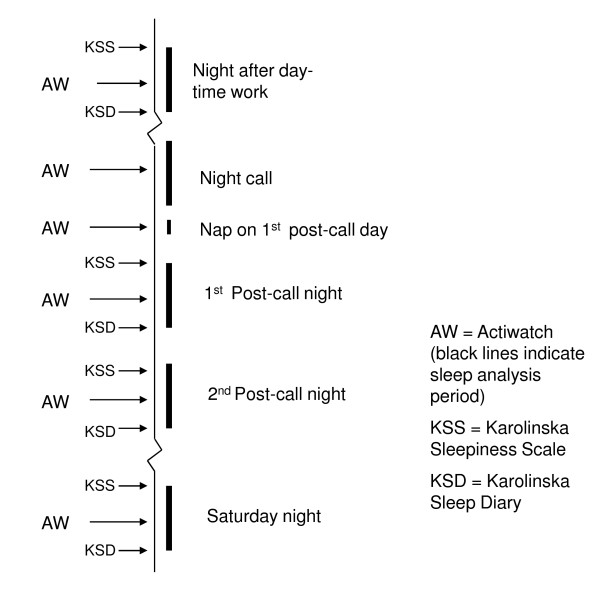
**Flowchart for sampling of data**.

Analyses of total sleep duration and sleep efficiency were performed, and comparisons were made between one night after daytime work, one night call, the following first and second nights post-call, and Saturday night for every participant (see Figure 1). These nights were determined a priori. However, for practical reasons the participants wore AW during the whole study period. For those who had more than one night-call duty in the same week during the monitoring period, the last one of these was chosen for analysis. To compensate for the possible effect of habituation to the sampling procedures, the days used for the analyses were selected in a counter-balanced design. Accordingly, the first occasion of sampling was equally distributed between ordinary work days, days related to night-call duty, and Saturdays. A control was added to ensure that the ordinary workdays and Saturdays included in the statistical analysis were always at least three days from a night call. The repeated measures made it possible for participants to serve as their own controls in the comparisons between days.

### Statistics

The statistical computations were made using SPSS 15.0 (SPSS Inc, Chicago, IL). P-values below 0.05 were considered statistically significant. For the comparison across days a repeated measures model was specified, using the linear mixed models module of SPSS. The AW sleep variables were normally distributed in the majority of subgroups. KSD data were ordinal scale data, but the scores still showed a fairly normal distribution. The independent variables were *Day *(5 levels: [i] night after day-time work; [ii] night call; [iii] 1^st ^post-call night; [iv] 2^nd ^post-call night and [v] Saturday and S*pecialist Group *(2 levels: ANEST vs. PENT), which was treated as a between-group factor. The dependent variables were the AW, KSS, and KSD variables. Gender and age were entered as covariates. Only age yielded a substantial impact (p < 0.2) and only on the AW measures. For this reason age was included as a covariate when analysing the AW measures [29,30].

The statistical modelling included the two-way interaction between Day and Group. However, the interaction term was not significant for any of the dependent variables (p = 0.1-0.8). The choice of model was governed by Schwarz's Bayesian Criterion (BIC) and a first order autoregressive (heterogeneous) covariance structure (ARH1) was applied, which showed the best fit. Residual analysis showed no outliers or marked deviations from normality assumptions. A univariate analysis using the General Linear Model module was used for testing group differences in the cross-sectional KSQ data and in comparisons between day sleep and no day sleep post-call. Because of the risk of obtaining biased results when applying parametric methods to ordinal data, or to data with skewed distributions, the outcome of AW, KSS, and KSD measures were also checked using nonparametric methods, which do not rely on assumptions. Specifically, Friedman's two-way analysis of variance test for overall testing of several related samples, Wilcoxon's Matched Pairs Signed Rank Test, as well as the Sign Test in comparisons of two related samples, and Mann-Whitney U test for two unrelated samples were used. However, the non-parametric analyses gave largely the same results as the mixed models analyses. Because the non-parametric analyses in the SPSS software do not allow for covariate inclusion, or the modelling of correlation between repeated measures, the parametric method was the preferred method in the end.

## Results

### Actigraphy

No interactions were found between Group and Day. For total sleep time and sleep efficiency there were main effects of Day (p < 0.001 and p = 0.045 respectively, Tables 2 and 3). Difference in total sleep time between nights ranged from 35-233 minutes, but the sleep efficiency differed by only 2-5%. The shortest sleep was recorded on night call, and a short sleep was also recorded on the night after daytime work when compared with both the two nights post-call and the Saturday night. Four participants in the ANEST group did not sleep at all during the night call. As might be expected, the sleep efficiency for all the other participants was somewhat lower on night call than on either the first or second post-call nights, but not different from the night after daytime work or Saturday night. As part of the recovery after night call, some participants (14/15 for ANEST, 8/17 for PENT) took a daytime nap (mean 2:30 hours, SD 1:27 hours) after their night call. An additional analysis of the effect of this on the following night's sleep showed, as expected, that those who took a nap had a shorter sleep the following night (p = 0.04). The difference disappeared when daytime sleep duration was included as a covariate (p = 0.88). Note that the daytime nap was presented separately from the sleep during first post-call night, but they both represent recovery sleep after night call.

**Table 2:** Crude data of sleep monitored by Actiwatch in anaesthesiologists (ANEST) and paediatricians and ENT-surgeons (PENT).

	ANEST (n = 15)		PENT (n = 17)	
	Mean	SD	Mean	SD
*Day-time work*				
Sleep start	23:19	01:11	23:48	00:57
Sleep end	06:02	00:24	06:38	00:35
Total sleep time (hours and minutes)	06:01	1:04	06:06	0:46
Sleep efficiency (%)	90	5	89	6
*Night call*				
Sleep start	02:53	01:26	03:15	01:44
Sleep end	06:44	00:54	07:39	00:40
Total sleep time (hours and minutes)	02:22	01:48	03:42	01:36
Sleep efficiency (%)	89	10	85	8
*1^st ^post-call night*				
Sleep start	23:35	01:12	23:29	01:35
Sleep end	06:35	00:38	07:52	01:28
Total sleep time (hours and minutes)	06:15	01:01	07:37	01:50
Sleep efficiency (%)	90	4	91	4
*2^nd ^post-call night*				
Sleep start	00:10	01:27	00:18	01:25
Sleep end	07:06	01:09	07:56	01:09
Total sleep time (hours and minutes)	06:16	01:19	06:55	01:08
Sleep efficiency (%)	91	3	91	4
*Saturday (off duty)*				
Sleep start	23:42	00:44	00:53	01:18
Sleep end	07:28	01:00	08:35	01:25
Total sleep time (hours and minutes)	06:48	00:46	06.56	00:54
Sleep efficiency (%)	88	5	90	4
	**ANEST (n = 14)**		**PENT (n = 8)**	
		
	**Mean**	**SD**	**Mean**	**SD**
		
*Day-time nap post-call*				
Sleep start	11:53	01:52	13:51	03:35
Sleep end	14:47	02:24	15:54	03:29
Total sleep time (hours and minutes)	2:52	01:27	01:50	01:17
Sleep efficiency (%)	91	4	91	4

**Table 3:** Comparison between days for all participant's Actiwatch night sleep measures (n = 32), KSS^2 ^(n = 29) and KSD^3^, (n = 32) ratings.

	Night after day-time work		Night call		1^st ^post-call night		2^nd ^post-call night		Saturday night		Type III F-test
	Mean	95% CI	Mean	95% CI	Mean	95% CI	Mean	95% CI	Mean	95% CI	P-value
AW sleep measures											
Total sleep time (min)	364^a^	344-383	186	148-224	419^b^	385-452	399^b^	373-425	412^b^	393-431	< 0.001
Sleep efficiency^1 ^(%)	90	88-92	86^c^	83-90	90	89-92	91	90-92	89^d^	88-91	0.045
											
KSS measures^2^											
Sleepiness (mean)	5.6	4.9-6.2	---		6.1	5.4-6.8	5.5	4.8-6.3	5.1	4.2-6.0	0.206
Mental fatigue	6.1	5.6-6.6	---		7.1^e^	6.5-7.7	6.1	5.4-6.9	5.8	5.0-6.7	0.004
											
KSD measures^3^											
SQI^4^	4.2	3.9-4.4	---		4.3	4.1-4.5	4.3	4.0-4.5	4.2	3.9-4.4	0.673
Sufficient sleep	3.0	2.7-3.4	---		2.9	2.5-3.3	3.1	2.7-3.6	3.6	3.2-4.0	0.052
Ease awakening	2.6^f^	2.2-3.0	---		2.8^f^	2.5-3.2	3.0	2.6-3.4	3.4	3.0-3.8	0.042
Well rested	2.6^f^	2.2-3.0	---		2.4^g^	2.0-2.8	3.0	2.6-3.4	3.3	2.9-3.7	0.013

(Estimated means are presented).

Note: 1. Sleep efficiency = the relative sleep time between sleep start and sleep end in %, (with awakenings during the night accounted for).

2. KSS (Karolinska sleepiness scale)-ratings are 1-10 (low-high). 3. KSD (Karolinska sleepiness diary)-ratings are scored 1-5, where 5 is the most positive. 4. SQI = (Sleep quality index) 1-5 (low quality-high quality), is the mean of 4 variables in the KSD.

^a ^Higher than night call, but lower than 1^st ^and 2^nd ^post-call nights and Saturday night (P's < 0.05). ^b ^Higher than night after day-time work and night call (P's < 0.05). ^c ^Lower than 1^st ^and 2^nd ^post-call nights. ^d ^Lower than 2^nd ^post-call night (P's <0.05). ^e ^Higher than daytime work, 2^nd ^post-call night, and Saturday. (P' < 0.01). ^f ^Lower than Saturday night (P's < 0.05). ^g ^Lower than Saturday night and 2^nd ^post-call night (P's < 0.05).

### Log-book

The participant's notes showed that 50% in the ANEST group got one or two emergency alarms (cardiac arrest, major trauma or likewise) during the night call, but there were none for the PENT group. The ANEST group reported more frequent involuntary awakenings for patient consultations after bedtime (usually 02:00 h or later). In addition, four participants in the ANEST group did not get the opportunity to sleep at all during night call. In the ANEST group 93% reported a nap on the first post-call day and in the PENT group 47%, which is in accordance with the sleep data in table 2. Only one of the ENT surgeons reported an acute operation during night-call.

### Rating scales

There was no interaction between Group and Day in the self-reported KSS and KSD measures. The KSS sleepiness score at bedtime did not differ between days; however mental fatigue was significantly higher on the first post-call evening compared with the other days (1.0-1.3 difference in scores). The KSD scores indicated a feeling of being less well rested on the morning after the first post-call night, compared with the mornings after the second post-call and Saturday nights (0.6-0.9 difference in scores), but not different from the morning after ordinary daytime work. There was no difference between the mornings after the second post-call night and Saturday night. No effect of Group was found for any of the KSS or KSD variables (Table 3). In the KSQ sleep indices there was no difference in habitual sleep between physician groups (p > 0.5). The respective scores (mean [SD]) for the ANEST and PENT groups were 3.6 (1.0) and 3.8 (0.5) for disturbed sleep, 3.7 (0.8) and 3.6 (0.7) for the sleepiness index, and 3.0 (0.9) and 3.0 (0.8) for the awakening index.

## Discussion

The present study aimed to examine whether a 16-hour night-call schedule negatively affected recovery in the ANEST group compared with the PENT group. The results showed that, in terms of subjective scores, the main sleep restitution was completed for both physician groups in the first 24 hours after night call. However a full recovery required an additional 24 hours. This was indicated when the scores for being "well rested" had reached the same levels as after a Saturday night's sleep. Previous studies of shift work have shown that recovery sleep after a night shift is normally extended by only one or two hours [31]. Experimental studies of moderate sleep deprivation (≈24 hours time awake) have consistently shown that recovery sleep contains a higher amount of slow-wave sleep (SWS), whereas the increase in sleep duration is limited [32]. The temporary increase in SWS is regarded as the core component of recovery during sleep [33]. Accordingly, as long as sleep deprivation is not too severe, the temporary increase in SWS may be sufficient for biological restoration. Thus the few extra hours of sleep after night duty observed in the present study are probably compatible with sufficient sleep recovery. According to recent comprehensive reviews, even a severe sleep debt might be overcome by a modest recovery sleep [34]. However, the fact that the total sleep during not only the first post-call night, but also the second post-call night, was longer than sleep after normal daytime work could indicate that there was still some sleep deficit left to recover on the second night post-call. There were no significant group differences in either subjective reports of sleep and recovery from night call or objective sleep measures. This could be interpreted to mean that work on call is equally demanding for all the participating physician groups. However, there could be selection bias in physicians' choice of speciality, such that, as a group, those who choose anaesthesiology may be more resilient following on-call work and sleep loss. This is of course merely speculative and so far not confirmed in the scientific literature. Indeed there is reported to be a genetic polymorphism concerning resistance to prolonged wakefulness in the healthy population. However physicians do not seem to be overrepresented in the resistant group [35]. Indeed, even in a group of jet fighter pilots there was a systematic inter-individual difference in performance after sleep loss [36]. It may even be the case that work characteristics/demands are not the major determinants of changes in sleep and recovery in the studied physician groups.

As expected, the general subjective sleep quality according to KSQ was good and corresponded to a level usually found in a healthy population [37]. The differences in subjective sleep quality measured using KSD were relatively small, albeit significant for some of the variables. Thus, ratings of feeling refreshed from sleep (well rested) at awakening after the first night of sleep after night call and on mornings after daytime work were similar. However, two days after night call the scores were similar to Sunday morning (i.e. morning after Saturday, in the tables), which is interpreted as a reasonable level of full recovery from night call. The between-day differences in morning scores on KSD variables seemed to correspond to the patterns of mental fatigue in KSS in the preceding evenings, both indicating that two nights' sleep was needed for full recovery after night call. The KSS sleepiness scores followed the same pattern, but there was no statistical difference between days. The present findings are in accordance with previous shift work studies, where recovery from a night shift with moderate disturbance of circadian rhythms requires two nights' sleep [38]. This also corresponds closely to the subjective reports from the participants concerning estimated time needed for recuperation after night call in general.

The fact that the physicians did not report any problems with insomnia or sleepiness speaks against the present working schedules causing any severe adverse effects on sleep in general. However, the previous polysomnographic study of physicians on call showed a preserved amount of deep sleep during call, but a large loss of REM (rapid eye movement) sleep [7]. This is also a well-known pattern from experimental studies of shift work [39]. Even though the SWS was probably sufficiently recovered, some adverse effects of insufficient REM sleep in the present participants cannot be ruled out. Nevertheless, the unexpected finding of short sleep after their ordinary work days is more troubling. Hence, even though sleep quality and sleep efficiency were sufficient in the whole group of physicians, they may still have a general sleep deficiency, which may constitute a health risk in a long-term perspective. There are strong indications of an elevated risk of diabetes and myocardial infarction in short sleepers (5-6 hours) [40,41]. Too short or too long sleep has also been associated with higher mortality, and according to recent studies, 7 to 8 hours of night sleep seems to be optimal for long-term survival [42]. In fact, an epidemiological study of Swedish anaesthesiologists indicated higher mortality compared with other specialists, but this was not confirmed by other Scandinavian studies [43,44]. However, the sleep duration of only 6 hours after daytime work found in the present study seems to represent a chronic sleep deficit of 1.5 hours in view of subjective reports of a mean need for 7.5 hours of sleep, and may therefore constitute a health risk. Despite different methods of attaining sleep measures, it is interesting to compare the total sleep times based on AW in the present study with the sleep times measured using EEG in the study by Åkerstedt et al [7]. In that study, which had a design similar to the present study, physicians monitored by ambulant EEG had roughly the same sleep duration on call as our participants, but about 1 hour longer sleep on post-call recovery and after ordinary daytime work, and a somewhat shorter daytime nap post-call. In a study of internal medicine residents there was no difference in sleep duration by actigraphy on postcall nights compared with non-call nights [45]. However, in this study the length and starting point of the night shift and other characteristics of the night-call were not clearly accounted for. This made it difficult to compare with the results in our present study. Different length of the night call, absence of naps and of experienced specialist physicians in the Saxena study might explain the divergent results concerning recovery.

A major strength of the present study is the use of multiple kinds of measures and types of data, such as global and real-time self-report measures, as well as objective sleep registrations. Another strength is the repeated measures over several days using the subjects as their own controls. This design made it possible to follow the dynamics of the recovery pattern. As verified by the participants' logbooks concerning special circumstances during the AW-registration, the specific days that were analysed seemed fully representative of the whole period of days monitored. For this reason we do not believe that any "extreme" days can explain the results.

One limitation, common in observational studies taking the present approach, is the limited sample size, because for practical reasons it is difficult to carry out this type of study with a larger group. Moreover, we did not have any precise measure of individual workload or of leisure activities that might influence sleep duration and quality during the days analysed. Because of the long period of data collection it was not realistic to demand an extremely detailed information each day and night. However, there were strong indications in the personal log-books of a heavier work load for ANEST compared with PENT during night call duty. In general there were no group differences with respect to overtime work, family situation, or worries over family matters, and there were no reports of any extraordinary loads or adverse events for the participants during the monitored period. Concerning sports activities during leisure time, the reports did not differ between groups.

## Conclusions

Despite considerable sleep loss during work on night call, the physicians' self-reports indicate that they recovered after two nights' sleep. We conclude that these 16-hour night duties were compatible with sufficient short-term recovery in both physician groups, but the limited sleep duration in general still implies a long-term health concern. These results may contribute to the establishment of safe working hours for night-call duty in physicians and other health-care workers.

## Competing interests

The authors declare that they have no competing interests.

## Authors' contributions

BM performed the data collection and statistical analyses, wrote the manuscript and made substantial contributions to the design of the study. GK contributed to the design of the study and interpretation of data, and commented on the manuscript. BK contributed to the interpretation of data, statistical analyses and commented on the manuscript. RP contributed to the conception and design of the study, and commented on the manuscript. PF contributed to the conception of the study and commented on the manuscript. PØ conceived of the study, supervised the design and commented on the manuscript. All authors have read and approved the final manuscript.

## Pre-publication history

The pre-publication history for this paper can be accessed here:

http://www.biomedcentral.com/1472-6963/10/239/prepub
